# A46 METABOLIC OUTCOMES 12-MONTHS POST-BARIATRIC SURGERY: SLEEVE VERSUS ROUX-EN-Y GASTRIC BYPASS

**DOI:** 10.1093/jcag/gwad061.046

**Published:** 2024-02-14

**Authors:** S Bharatselvam, K Schwenger, Y Ghorbani, S Fischer, T Jackson, A Okrainec, J P Allard

**Affiliations:** . University Health Network, Toronto, ON, Canada; . University Health Network, Toronto, ON, Canada; . University Health Network, Toronto, ON, Canada; . University of Toronto Department of Laboratory Medicine and Pathobiology, Toronto, ON, Canada; . University of Toronto Division of General Surgery, Toronto, ON, Canada; . University of Toronto Division of General Surgery, Toronto, ON, Canada; . University of Toronto Department of Medicine, Toronto, ON, Canada

## Abstract

**Background:**

Obesity and its related comorbidities, such as type 2 diabetes (T2D), hyperlipidemia, cardiovascular disease (CVD), and non-alcoholic fatty liver disease (NAFLD) are on the rise. When lifestyle modifications, such as diet and exercise, are ineffective at producing sustained weight loss and improvements in obesity-related comorbidities, other interventions may be recommended. Bariatric surgery is considered in those who have a body mass index (BMI) over 40 kg/m2 or with comorbid conditions and a BMI between 35-40 kg/m2. Two commonly used procedures within this category of surgery are Roux-en-Y gastric bypass (RYGB), the gold standard for weight loss; and laparoscopic sleeve gastrectomy (LSG). Though both surgeries are effective at inducing weight loss, more information is needed on their comparative effectiveness in improving clinical and biochemical outcomes in patients with T2D, hyperlipidemia, and NAFLD in real-world practice.

**Aims:**

Therefore, our objective was to determine the overall effectiveness of RYGB versus LSG at 12-months post-surgery and specifically assess their responses in those with hyperlipidemia, T2D and NAFLD. We chose to analyze both glucose and lipid parameters along with anthropometric measures as they not only provide insight into improvements in comorbid outcomes of obesity but also little-to-no prospective studies compare all of these outcomes in one study using the same cohort.

**Methods:**

This is a prospective cross-sectional and cohort study of 142 patients who underwent bariatric surgery. Clinical and biochemical data were collected at baseline, prior to surgery and 12-months post-bariatric surgery. A liver biopsy was collected during surgery to diagnose NAFLD. The main outcome was 12-month changes in lipid parameters, mainly total cholesterol, between types of surgery.

**Results:**

107 individuals underwent RYGB (75.4%) and 35 underwent LSG (24.6%). Both groups were similar at baseline except for the higher waist circumference and proportion of males undergoing LSG. At 12-months post-surgery, RYGB versus LSG resulted in a significantly lower BMI (pampersand:003C0.0001), triglycerides (pampersand:003C0.05), total and LDL cholesterol (pampersand:003C0.005). Interestingly, ALT was significantly lower in those who underwent LSG (pampersand:003C0.005). In subgroup analysis for determining the effect of the type of surgery in those with comorbidities of obesity, RYGB was superior at improving lipid-related parameters in those with hyperlipidemia with a significant decrease in total cholesterol -0.48 (-0.84, 0.34) (pampersand:003C0.05) whereas LSG was superior at reducing ALT in those with NAFLD with significant decreases in total cholesterol and LDL.

**Conclusions:**

RYGB leads to greater reductions in BMI and lipid parameters, especially in those with hyperlipidemia, whereas LSG showed greater improvements in liver enzymes in those with NAFLD.

**Funding Agencies:**

CIHR

